# At the Mercy of Strategies: The Role of Motor Representations in Language Understanding

**DOI:** 10.3389/fpsyg.2013.00027

**Published:** 2013-02-04

**Authors:** Barbara Tomasino, Raffaella Ida Rumiati

**Affiliations:** ^1^Istituto di Ricovero e Cura a Carattere Scientifico “Eugenio Medea”San Vito al Tagliamento, Italy; ^2^Neuroscience Area, Scuola Internazionale Superiore di Studi AvanzatiTrieste, Italy

**Keywords:** motor simulation, word representations, action understanding, imagery, cognitive strategies

## Abstract

Classical cognitive theories hold that word representations in the brain are abstract and amodal, and are independent of the objects’ sensorimotor properties they refer to. An alternative hypothesis emphasizes the importance of bodily processes in cognition: the representation of a concept appears to be crucially dependent upon perceptual-motor processes that relate to it. Thus, understanding action-related words would rely upon the same motor structures that also support the execution of the same actions. In this context, motor simulation represents a key component. Our approach is to draw parallels between the literature on mental rotation and the literature on action verb/sentence processing. Here we will discuss recent studies on mental imagery, mental rotation, and language that clearly demonstrate how motor simulation is neither automatic nor necessary to language understanding. These studies have shown that motor representations can or cannot be activated depending on the type of strategy the participants adopt to perform tasks involving motor phrases. On the one hand, participants may imagine the movement with the body parts used to carry out the actions described by the verbs (i.e., motor strategy); on the other, individuals may solve the task without simulating the corresponding movements (i.e., visual strategy). While it is not surprising that the motor strategy is at work when participants process action-related verbs, it is however striking that sensorimotor activation has been reported also for imageable concrete words with no motor content, for “non-words” with regular phonology, for pseudo-verb stimuli, and also for negations. Based on the extant literature, we will argue that implicit motor imagery is not uniquely used when a body-related stimulus is encountered, and that it is not the type of stimulus that automatically triggers the motor simulation but the type of strategy. Finally, we will also comment on the view that sensorimotor activations are subjected to a top-down modulation.

## Introduction

Functional magnetic resonance imaging (fMRI) studies have identified regions in sensorimotor cortex that are activated preferentially by action-related words but also for words with no motor content. The extent to which these patterns of activation are modulated by bottom-up or top-down mechanisms is currently unknown. Many cognitive processes rely on both “bottom-up” and “top-down” processing. One example is found in the mental rotation domain in which bottom-up processing is first triggered by the stimulus category, and then continues until sensorimotor or visuospatial operations are engaged. On the other hand, top-down processing refers to the modulatory effect exerted by cognitive strategies, which can be implicitly adopted by participants while solving the task at hand. Accordingly, motor representations can or cannot be activated depending on the type of strategy the participants adopt to perform tasks involving bodily – and non-bodily related stimuli. Whether the same pattern, speaking for a top-down modulation of sensorimotor activation depending on strategy (or contextual) factors might be applied to the action-related word processing domain is currently under debate. Despite growing research efforts, the actual *cause* of the observed motor system activity during action word processing remains elusive (Kemmerer and Gonzalez-Castillo, 2010). Some authors argue that the action-related aspects of a word’s meaning are represented in and around the motor strip and that these regions are automatically and invariably activated when action words are encountered, and should not be modulated by attentional demands, i.e., associationist theory (Pulvermuller et al., 2001, 2005a; Pulvermuller, 2005). Sensorimotor activations observed during language processing reflect how word meaning is stored in the brain. According to embodied theories of cognition, sensory-motor systems play an important role in the representation of concepts (Lakoff, 1987; Glenberg, 1997; Barsalou, 1999; Lakoff and Johnson, 1999; Glenberg and Kaschak, 2002; Feldman and Narayanan, 2004; Gallese and Lakoff, 2005). A bold version of embodiment theory (Gallese and Lakoff, 2005) does not just assume that our concepts can be represented in sensorimotor systems but rather that they *are* the sensorimotor systems. Secondary, embodied proposals argue that word meaning is linked to sensorimotor experience derived from the motor and perceptual simulation during comprehension; however, these simulation processes are not a reflection of how meaning is represented (Mahon and Caramazza, 2008). Versions of embodied theories (Barsalou et al., 2008; Borghi and Cimatti, 2009; Dove, 2009; Louwerse and Jeuniaux, 2010; Borghi, 2012) based on a mixed view of how concepts are represented, propose that both amodal and modal conceptual representations coexist in conceptual processing, i.e., a “representational pluralism”; they also extend the embodied view of cognition to account not only for language grounding but also for the social and normative aspects of cognition (Borghi and Cimatti, 2009; Wilson-Mendenhall et al., 2012). However, it is still not clear how these recent theoretical developments can account for the lack of sensorimotor activation in some of the action-related word processing studies. Although simulation and associative learning theories are difficult to tease apart (e.g., Keysers and Perrett, 2004; Brass and Heyes, 2005), the contribution of the top-down strategic modulation might be a promising approach to investigate the interaction between the language and motor systems. With respect to whether the motor areas activation is bottom-up, both the embodied cognition theory and associationist theory lead to identical predictions. On both accounts the activation of the sensorimotor areas observed in several fMRI studies investigating action-related word processing is maintained to be stimulus-dependent (Hauk et al., 2004; Pulvermuller, 2005; Ruschemeyer et al., 2007; Kemmerer and Gonzalez-Castillo, 2010). Thus different mental operations may be at work during language processing depending on whether the stimulus type is an action-related word or a non-action word. By contrast, the associationist theory is certainly not in agreement with the top-down hypothesis. According to the associationist theory (Pulvermuller et al., 2001, 2005a; Pulvermuller, 2005), the activation of the sensorimotor cortex “should not require people to attend to language stimuli, but should instead be *automatic*” (Pulvermuller et al., 2005b). The top-down hypothesis instead holds that motor activation is not automatically triggered by the type of stimulus but by the type of strategy. Also the embodied cognition hypothesis, as it claims that “understanding” *is* sensory and motor simulation, is not compatible with the view that the type of strategy selected depends on top-down modulation of the context and tasks demands. Rather the top-down hypothesis is in line with the disembodied view the motor system may be activated but not necessarily so (Mahon and Caramazza, 2005, 2008).

Although previous studies also point to an involvement of the premotor cortex (PM) in processing action verbs (e.g., Tettamanti et al., 2005, 2008; Aziz-Zadeh et al., 2006), in the present review article we were primarily interested in the neural response pattern of the (left) M1 cortex, given the susceptibility of this region to top-down modulation (e.g., Kosslyn et al., 2001).

## A Lesson from Mental Rotation

### Bottom-up hypothesis

Bottom-up and top-down processes have specifically been triggered in studies that aimed at investigating whether the recruitment of motor representations in mental rotation depends on the stimuli, or on the particular mental operation adopted in solving the task, respectively. Although mental rotation (hereafter MR) is generally held to be under conscious control (Cooper and Shepard, 1973), there is also evidence that part of the processing escapes awareness. One way to go about characterizing the MR operations is to evaluate whether they differentially respond to the type of stimulus that is mentally rotated (Kosslyn et al., 1998; Rumiati et al., 2001; Tomasino et al., 2003), the reference frame (Wraga et al., 1999; Zacks et al., 1999, 2002), or to the type of strategy (Kosslyn et al., 2001).

The standard view has long maintained that the mechanisms involved in MR were essentially bottom-up, that is externally triggered by low-level information derived from the stimuli to be rotated (we will refer to this as the type of stimulus hypothesis). Three are the types of stimulus used in MR experiments: the 2D alphanumeric characters, 3D abstract pictures such as cubes and body parts such as hand shapes. All these stimuli can elicit two types of MR mechanisms (Kosslyn et al., 1998): (i) object-based spatial transformations, and (ii) egocentric perspective transformations. The former MR mechanism generates simulated rotations of, for instance, hands, remodeled as reaching movements in which subjects implicitly turn their own hands into correspondence with the pictured hand stimulus (Parsons et al., 1995, 1998; Kosslyn et al., 1998; Parsons and Fox, 1998). By contrast, the latter mechanism corresponds to imagined movements of one’s point of view, generally used for mentally rotating external abstract pictures in the visual space, without the need of a motor simulation (Zacks et al., 2002, 2003). Thus, different operations may be recruited in MR depending on whether the stimulus type is a body part or a two or three-dimensional object (Parsons et al., 1995, 1998; Kosslyn et al., 1998; Parsons and Fox, 1998).

Neuropsychological studies documented a double dissociation between the processes underlying these two types of transformations, each of which can be selectively affected as a result of brain damage. In a group study, patients with right brain damage (RBD) showed impaired MR of external objects (e.g., a puppet and flag shapes), while patients with left brain damage (LBD) showed impaired MR of hands (Tomasino et al., 2003). These results are compatible with single case reports. On the one hand, patient MT, with left hemisphere brain damage, was described as being selectively impaired at left or right hand decisions despite being still able to mentally rotate Shepard and Metzler’s stimuli (Rumiati et al., 2001). On the other hand, patient JB, with a bilateral inferotemporal lesion, was also observed as having a deficit in performing MR of Shepard and Metzler’s stimuli (Sirigu and Duhamel, 2001). However, the ability to mentally rotate motor images of body parts was not investigated in JB or in other posterior left (Kosslyn et al., 1985; Metha and Newcombe, 1991; Morton and Morris, 1995) or right (Ratcliff, 1979; Farah et al., 1988; Ditunno and Mann, 1990; Bricolo et al., 2000) brain-damaged patients with a MR deficit.

Consistently with the notion that MR operations interact with the type of stimulus, neuroimaging research has provided *in vivo* evidence that different types of stimuli trigger different mental rotation-related clusters (Kosslyn et al., 1998). Using the Positron Emission Tomography (PET), these authors monitored the regional cerebral blood flow (rCBF) of healthy subjects during two mental rotation tasks. In the first task, subjects compared and decided whether two angular branching forms (i.e., Shepard–Metzler cubes) had the same (baseline) or different orientations (rotation condition), while in a second task stimuli used were line drawings of hand shapes. Mentally rotating branching forms enhanced bilateral activation in the right parietal lobe and in Brodmann Area (BA) 19, whereas mentally rotating hands enhanced unilateral left activation in the precentral gyrus (M1), most of the parietal lobe, the primary visual cortex, the insula, and frontal BAs 6 (PM) and 9 (superior frontal cortex). Kosslyn et al. (1998) proposed that at least two independent mechanisms are engaged in the mental rotation of hands and objects, one requiring processes that prepare motor movements, and one that does not, and that motor processes are recruited only when participants mentally rotated hands but not when they mentally rotated Shepard and Metzler’s stimuli.

Psychophysical evidence too demonstrated that hands are a special type of stimulus. Response times during MR of body parts reflect the degree of awkwardness associated to the orientation of the hand stimulus and the length of the imagined path (Parsons, 1987, 1994; Parsons et al., 1995). This reaction time (RTs) pattern provides the evidence that subjects imagine a spatial transformation of their own body part from its actual orientation until it matches the stimulus orientation. By contrast, the effect of biological constraints on RTs has never been found during MR of external objects, thus suggesting that MR may recruit different mechanisms depending on the type of stimuli involved in the mental transformation. Accordingly, MR of hands, but not of objects, implicitly triggers sensorimotor imagery rather than visuospatial imagery alone.

The view that MR operations are differentially triggered depending on the *type of stimulus* to be rotated, as suggested by the above reviewed studies, was soon modified following a neuroimaging study (Kosslyn et al., 2001) in which it was argued that the left M1 was not recruited for mentally rotating only body parts such as hand shapes, but also non-body-part stimuli such as external abstract objects (Cohen et al., 1996; Tagaris et al., 1996; Richter et al., 1997; Carpenter et al., 1999; Lamm et al., 2001; Vingerhoets et al., 2001), even though subjects were not explicitly instructed to use a particular strategy (Kosslyn et al., 2001). Kosslyn et al. (2001) argued that subjects might have spontaneously adopted a motor strategy, accounting thus for these results. This (Kosslyn et al., 2001) and other studies (Wraga et al., 2003; Tomasino and Rumiati, 2004; Tomasino et al., 2004) that soon followed paved the way to the formulation of the top-down hypothesis, as we will discuss in the following section.

### Top-down hypothesis

According to the top-down hypothesis, higher-level mechanisms guide individuals to select the most suitable cognitive strategy that allows them to solve MR tasks. Thus the original view that different MR mechanisms are elicited depending on the type of stimulus under rotation, has later been replaced by the hypothesis that this selection mechanism rather depends on the frame of reference or the type of strategy used in imagining inanimate objects rotating (Kosslyn et al., 2001; Zacks et al., 2002, 2003). This *top-down hypothesis* holds that there could be at least two strategies involved in MR. One strategy encompasses imagining what one would see if he/she manipulates an object, the other implicates imagining what one would see if someone else, or an external force, manipulates an object (Kosslyn et al., 2001). In that PET study (Kosslyn et al., 2001), subjects mentally rotated Shepard and Metzler stimuli using either an external strategy or an internal strategy. Before performing this MR task, subjects either viewed an electric motor device rotating the 3D cube (external action) or they rotated it manually (internal action). Afterward, subjects performed the MR by imagining grasping the object, and turning it with their own hand, or by mentally viewing the stimulus as if it were being rotated by an electric motor device. The same region that in Kosslyn et al.’s (1998) PET study was activated in association with MR of hands only – the left primary motor cortex – here was enhanced when subjects simulated a manual rotation of the Shepard and Metzler’s stimuli.

Neuropsychological evidence further supported the view that what matters in MR is the type of strategy adopted (Tomasino and Rumiati, 2004). Patients with unilateral brain lesions and healthy control subjects were instructed to adopt a motor (egocentric transformation) and, in a different block, a visual strategy (allocentric transformation) when performing MR of hand shapes (Experiment 1) or Shepard and Metzler’s stimuli (Experiment 2). Independent of the type of stimulus, LBD patients showed a selective deficit in MR either hands and 3D cubes as a consequence of their manual activity, whereas RBD patients performed pathologically on a MR task in which they were required to apply a visual strategy (Tomasino and Rumiati, 2004). This study showed how MR could be achieved by recruiting different strategies, implicitly triggered or prompted at will, and each sustained by a unilateral brain network.

How can we reconcile the neuropsychological findings, supporting the view that MR is a lateralized process which depends on the type of stimulus (Tomasino et al., 2003), with those in favor of MR as depending on the strategy adopted (Kosslyn et al., 2001; Tomasino and Rumiati, 2004)? While in Tomasino et al. (2003) LBD patients were impaired at mentally rotating hands but not external objects, and RBD patients showed the opposite pattern, in a subsequent study (Tomasino and Rumiati, 2004), LBD patients, explicitly encouraged to apply either the motor strategy or the visual strategy, failed to rotate both types of stimuli when the operation was solved by means of a motor strategy, but succeeded when the alternative visual strategy was selected. As Kosslyn et al. (1998) argued, in the absence of clear instructions, participants spontaneously adopt one or the other strategy to perform MR. According to whether the mental operation intrinsically requires imagining limb movements (somatomotor operation) or the motion of visual objects (visuospatial operation), MR can be solved via motor or visual strategy. Thus both bottom-up and top-down strategies are used in MR, and their selection seems to depend on task settings, instructions, and other variables. Participants may voluntarily adopt one or the other strategy if prompted by the experimenter but, in a free choice paradigm, the preferred strategy can also be stimulus-dependent. When subjects are not instructed to adopt a given strategy, the type of stimulus determines which one is going to be selected moreover, these strategies can be implicitly transferred from one type of MR to another, and lateralization might vary according to the order of block presentation (Wraga et al., 2003). Transcranial magnetic stimulation (TMS) studies have shown that stimulation over the left M1 slowed down MR of hands but not of letters (Tomasino et al., 2005) or feet (Ganis et al., 2000). In Tomasino et al.’s (2005) study, subjects were free to apply one or the other strategy, with the instructions requiring them to mentally rotate the stimulus on the right, and decide whether it was the same or a mirror image of the other. Since an interference effect due to stimulation was obtained only during MR of hands, it was held that hands *implicitly* require a mental motor transformation. By contrast, since TMS interferes with MR of hand shapes but not of letters, it has been argued that alphanumeric characters do not implicitly require a mental motor strategy (i.e., viewer-based) but rather a visuospatial strategy (i.e., object-based). Moreover, brain tumor patients with selective lesions, selectively affecting the hand sensorimotor representation, failed to mentally rotate hand shapes, but not letters, if they were free to use any cognitive strategy; this deficit, however, extended to abstract objects when the patients imagined moving them with their own hands, while maintaining the ability to visualize them rotating in space (Tomasino et al., 2010a). These neuropsychological findings provide conclusive evidence that discrete brain areas can be selectively recruited according to the strategy that is implicitly adopted while solving a cognitive task.

### Top-down modulatory effects in other cognitive domains

That partially discrete brain networks can support different cognitive operations depending on their purpose has been demonstrated in other cognitive domains. For instance, the visual information can be used either for identifying objects (along the “what” stream) or for guiding action (along the “how” stream; Milner and Goodale, 1995). These authors described a patient, DF, with visual form agnosia caused by a bilateral occipital lesion, as being severely impaired at perceptually judging the orientation of a line as well as at showing with her fingers the dimensions of objects that were visually presented; however, she was able to orient her hand in a posting task as well as to execute normal reaching-grasping movements (Goodale et al., 1991; Milner and Goodale, 1995). The opposite pattern was observed in patient RV, with a bilateral occipital lesion, who failed to grasp objects whose visual shape he was almost perfectly able to identify (Goodale et al., 1994).

The existence of different networks specialized in carrying out the same cognitive operation according to its purpose is supported by different sources of evidence. For instance, it has been shown that differential neural mechanisms were enhanced when subjects solved the line bisection task either manually (action) or as perceptual judgments (vision; Weiss et al., 2003). In particular, in the latter condition, a unilateral activation of the right inferior parietal cortex, anterior cingulate, dorsolateral prefrontal cortex, including also the extrastriate and superior temporal cortex bilaterally, was observed. By contrast, the manual bisection task enhanced activation in the extrastriate, superior parietal, and premotor cortices bilaterally.

Finally, it has been shown how hemispheric specialization might be dependent upon the nature of the task rather than on the nature of the stimulus (Stephan et al., 2003). In their fMRI study, 16 right-handed volunteers performed two different tasks on an identical set of four letter words, three of which written in black and either the second or third letter in red. While in the letter-decision task, the participants were asked to ignore the position of the red letter and indicate whether or not the displayed word contained the target letter “A,” in the visuospatial-decision task, they were required to ignore the language-related properties of the words and to judge whether the red letter was located left or right of the center of the word. Comparing letters in the visuospatial-decision task led to a significantly higher activation in the left inferior frontal gyrus, occipital cortex, ventral PM (PMv), anterior cingulate cortex (ACC), and supplementary motor cortex. In contrast, visuospatial decisions compared with letter decisions significantly increased the activation in the anterior and posterior parts of the right inferior parietal lobule. For the authors this functional dissociation suggests that the cognitive control mechanisms differentially directs attention to specific stimulus features and guide the subsequent information processing. When they analyzed the frontal regions responsible for cognitive control, an increased coupling between left ACC and left inferior frontal gyrus was found for letter decisions, and between the right ACC and right parietal areas for visuospatial decisions (Stephan et al., 2003). To conclude, the plasticity with which the brain adapts to the different tasks and contexts, and switches between hemispheres, in the studies reviewed above is comparable with the one found in the mental rotation domain (Tomasino and Rumiati, 2004).

## Mental Rotation and Action-Related Word Processing

### Bottom-up hypothesis

The recruitment of the sensorimotor areas observed in several fMRI studies investigating action-related word processing has been interpreted as being stimulus-dependent (Hauk et al., 2004; Ruschemeyer et al., 2007; Kemmerer and Gonzalez-Castillo, 2010). For example, lexical decisions about action verbs, i.e., to judge whether a verb is a real word or a pseudoword, were found to lead to stronger high-frequency EEG activity at recording sites located closely above primary motor (M1) cortex (Pulvermuller et al., 2001). Interestingly, action words related to different body parts, i.e., face, arm, or leg movements, compared with non-action words, activated the primary motor cortex and the PM in a somatotopic manner (Hauk et al., 2004; Buccino et al., 2005; Aziz-Zadeh et al., 2006). Listening to sentences expressing actions performed with the mouth, the hand, or the foot led to signal increased in different parts of the left PM depending on the effector involved in the action described in the sentence (Tettamanti et al., 2005; Aziz-Zadeh et al., 2006). TMS of the left M1 causes similar effector-specific M1 modulation during listening to hand and foot action-related sentences (Buccino et al., 2005), and during a lexical decision task (Pulvermuller et al., 2005b). In addition, the activation of the left M1 increased for action words (verbs and nouns) compared with non-action words (Oliveri et al., 2004).

Thus different mental operations may be at work during language processing depending on whether the stimulus type is an action-related word or a non-action related word. The sensorimotor activation during language processing has been interpreted as sensorimotor representations being an integral part of action word representation (Pulvermuller, 2005). According to the proponents of the associative learning approach (Pulvermuller, 2005), the activation of the sensorimotor cortex can play a specific functional role in recognizing action words (p. 578, Pulvermuller, 2005). Specifically, authors suggested that neurons in the fronto-central cortex differentially contribute to the semantic processing of action words, and hence called them semantic neurons, located in the inferior fronto-central cortex for face-related words, and in the superior central cortex for leg-related words (consistent with the known motor somatotopy; Pulvermuller, 2005).

A similar view is the one forwarded by the embodied hypothesis of language understanding according to which conceptual knowledge is grounded in sensory-motor systems (Barsalou, 1999; Feldman and Narayanan, 2004; Gallese and Lakoff, 2005). This idea is consistent with the view that word meaning is processed in dedicated cortical areas (e.g., Martin et al., 1995, 1996), and is in sharp contrast with the conceptual-level representation theory (e.g., Pylyshyn, 1984; Fodor, 2001), which suggests that the meaning of a verbally presented action is accessed through abstract amodal units. The latter view emphasizes the abstract, amodal, and symbolic character of concepts, which are thought to be represented outside the brain’s sensory-motor systems. According to this view, concepts are not represented within the sensory and motor systems – the (so-called) disembodied cognition hypothesis. According to the disembodied cognition hypothesis, conceptual representations are “symbolic” and “abstract” and, as such, qualitatively distinct. An intermediate position is represented by the secondary embodiment, according to which amodal conceptual representations are instantiated by retrieving sensory and motor information by an independent, but associated, semantic system (Mahon and Caramazza, 2008). Lastly, recent theories based on multiple types of representation (Barsalou et al., 2008; Borghi and Cimatti, 2009; Dove, 2009; Louwerse and Jeuniaux, 2010; Borghi, 2012) propose the existence of both amodal and modal conceptual representations in conceptual processing, i.e., a “representational pluralism” (Dove, 2009) they also extend the embodied view of cognition to account not only for language grounding but also for the social and normative aspects of cognition (Borghi and Cimatti, 2009; Wilson-Mendenhall et al., 2012). However, it is still not clear how these recent theoretical developments can account for the lack of sensorimotor activation in some of the action-related word processing. The view that sensorimotor areas are activated depending on the *type of word*, has been challenged by several studies which showed how the recruitment of the sensorimotor areas is not automatic as held before (Pulvermuller et al., 2005b), but rather context-dependent (Tomasino et al., 2007, 2008; Papeo et al., 2009; van Dam et al., 2010b, 2012; Willems et al., 2010).

### Top-down hypothesis

Similarly to what has been observed in the mental rotation domain, individuals might be using different strategies in trying to understand action-related words or phrases. One of these strategies involves implicit simulation, that is a process that occurs when subjects unconsciously simulate the movement while performing another task, even in the absence of a precise instruction to do so (Jeannerod and Frak, 1999). The tasks which have been found to elicit implicit simulation are: mental rotation of body parts (e.g., Zacks et al., 1999; Kosslyn et al., 2001), handedness recognition of a visually presented hand (e.g., Parsons and Fox, 1998), judgments as to whether an action would be easy, difficult, or impossible (Johnson-Frey et al., 2002), and recognizing and understanding actions of other individuals (e.g., Jeannerod and Frak, 1999). It has been suggested that implicit simulation activates effector-specific regions in the PM cortex, presumably because it facilitates further action planning whenever subsequent cues call for movements to be explicitly executed or to be imagined (Willems et al., 2010).

Consistently with the top-down hypothesis, when we are trying to understand action-related words may implicitly imagine the corresponding movement, thus triggering the underlying motor representation. In the mental rotation domain, it has been shown that if participants are not clearly instructed, it is the type of stimulus that determines which strategy will be selected (Wraga et al., 2003). In most of the fMRI experiments, evaluating the neural correlates of action-related language processing (e.g., Hauk et al., 2004; Buccino et al., 2005; Tettamanti et al., 2005), subjects were not instructed to explicitly imagine themselves or somebody else performing the movements. This, by itself, does not ensure that they might have nevertheless implicitly performed motor imagery. Thus, in the effort to control for putative motor imagery during word processing, participants were asked to perform an imagery task and a letter detection task with action and non-action verbs and found that, the imagery task compared to the letter detection task, led to an enhanced M1 activation for action verbs relative to non-action related (Tomasino et al., 2007). In other studies, the effector-specific activation of M1 was observed during semantic judgments on action verbs, relative to task conditions where the access to word meaning was less explicit or only incidental, e.g., letter detection or syllable counting (Papeo et al., 2009) or during imagery, but not during lexical decision of action-related stimuli (Willems et al., 2010), although authors found premotor activation during lexical decisions, consistently with results from a TMS study in which authors found that stimulation of hand-related PM modulated the processing of hand-related action verbs during lexical decisions (Willems et al., 2011). Evidence for such strategic effect has been recently found also on other brain networks during reading (Cummine et al., 2012).

According to the idea we are trying to put forward here, different task strategies cause participants to lean on different sensorimotor representations. In a series of studies investigating different aspects of language representations (e.g., morphology, grammar, category specificity, semantics), we checked the type of task used and whether M1 was explicitly reported among the activated areas in the critical comparisons involving action verbs.

On the one hand, we identified a series of studies involving action words or verbs in which no activation of M1 was found. For instance, Perani et al. (1999) used a *lexical decision* task involving concrete and abstract verbs (presented in their infinitive form) and nouns, and failed to find a selective activation of M1 when subjects processed concrete verbs (e.g., to brush, to comb, to write). Interestingly, making “*pleasant/unpleasant*” *decisions* about verbs and nouns, presented either as stem or inflected (e.g., for verbs: sing or sings), did not activate the M1 cortex for verbs relative to nouns (Longe et al., 2007). Neither did a task requiring *generating a verb* for a noun (Petersen et al., 1998). Other authors probed the *comprehension* of motion verbs and found (compared to pseudowords) stronger activity in the left ventral temporal-occipital cortex, bilateral prefrontal cortex, and caudate; however, there was no activation of M1 (Grossman et al., 2002). Furthermore, numerous neuroimaging studies found the middle/superior temporal gyrus to be activated during *action word generation* (Martin et al., 1995, 1996; Fiez et al., 1996; Tranel et al., 2005). Raposo et al. (2009), for instance, showed that *passive* *listening* to arm- and leg-related verbs, presented in isolation (e.g., *kick*), elicited M1 activation in study 1, whereas that literal sentences (as in “*kick the ball*”) and idiomatic sentences (as in “*kick the bucket*”), constructed using the same action verbs as in the single word study, elicited M1 to a lesser extent in study 2. Differently from passive listening of words presented in isolation, this latter task required participants to *listen to sentences* and to *decide* on half of them whether a visual *probe* word, presented on the screen a few seconds after the end of the sentence, *was related* to the meaning of the sentence. Interestingly, idiomatic sentences activated fronto-temporal regions, associated with language processing, but not motor and premotor cortices (Raposo et al., 2009). Passive listening and silent reading not always elicit M1 activation. *Passive listening* of action-related literal sentences, e.g., “biting the peach” as compared to metaphorical sentences including action words, e.g., “biting off more that you can chew,” did not elicit any significant activation of M1 (Aziz-Zadeh et al., 2006). Other authors instructed participants to *silently read* blocks of action words related to specific effectors (e.g., punch, bite, or stomp), and items with various levels of lexical information (non-body part-related meanings, non-words, and visual character strings presented in infinitive form) and, when a fixation cross or hashes were presented, to watch the stimuli without mentally reciting them (Postle et al., 2008). They failed to find a somatotopic organization of action-related language processing.

Other showed that *passive listening* to sentences describing actions performed with the mouth, the hand, or the leg, and to abstract sentences task (Tettamanti et al., 2005) activated the PM but not the M1. Other authors used a *silent reading* of sentences including manual action verbs plus a specific physical object presented in past, present, and future forms, as compared to abstract verbs, followed by a *reading comprehension* task, involving questions referred to a temporal aspect of the sentence (e.g., “Is the table currently being cleaned?”) in half of the cases and to a non-temporal aspect (e.g., “Did the sentence refer to a piece of furniture?”) in the remaining items. They found that irrespective of the tense, action-related sentences did not activate the M1 cortex (Gilead et al., 2013). In another fMRI study, participants *listened* *to* sentences including a hand/arm action verb (e.g., grab, punch), a verb primarily visual in nature (e.g., read, browse), and abstract verbs (e.g., allow, explain) and judged whether the sentences were sensible, pressing a response button with their left index finger only for sentences *judged to be nonsense* (Desai et al., 2010). M1 cortex was not reported among the activated areas neither for the motor vs. visual-related verbs contrast nor for the motor vs. abstract related verbs contrast. In addition, the overlap between areas activated in the motor localizer task and those activated in the motor vs. visual-related verbs contrast, motor vs. abstract related verbs contrast was found in the inferior postcentral focus (Desai et al., 2010). It has been shown that, while watching of short object-related action movies activated the hand sensorimotor area bilaterally, *listening to and producing* short sentences describing object-related actions and man-made objects did not (Tremblay et al., 2003). Tremblay and Small (2011) found a functional specialization within the PMv for observing actions and for observing objects, and a different organization for processing sentences describing actions and objects. In addition, the *generation* of verbs with strong motor association, in a *minimal phrase context* eliciting active semantic processing, as compared to a rhyming task, did not trigger activations in motor-related areas (Khader et al., 2010). Authors (Khader et al., 2010) reported stronger activation for verb generation in the left superior temporal gyrus. Other authors presented verbs denoting actions that one performs mostly with hands involved in a general motor program (e.g., to clean) or a more specific motor program (e.g., to wipe), plus as control 20 mouth-related words (van Dam et al., 2010a). Participants were instructed to *read* all words and perform *a categorization* task in which a go response should be made only to verbs denoting a mouth action. Van Dam et al. failed to report M1 cortex among the activated areas for the action-related vs. abstract verbs contrast, independent of whether actions were involved in a general motor program and more specific motor program. In another fMRI study by the same authors, participants were presented with (1) action words (i.e., words highly associated with a specific action, such as stapler), (2) color words (i.e., words highly associated with a specific color, such as wedding dress), and (3) action-color words (i.e., words highly associated with both an action and a color, such as tennis ball or boxing glove) and were instructed to *listen to* all words carefully and to perform a go/no-go semantic *categorization* task, in which go responses should be made only to words denoting objects that were associated with either a green color or a foot action (van Dam et al., 2012). These authors found that when participants were instructed to focus on the action performed on a word’s referent, as compared to when they were instructed to focus on the object’s color, no M1 activation was reported within action areas. In another study, subjects *listened* carefully *to indirect requests* (IRs) for action which are speech acts in which access to an action concept is required, although it is not explicitly encoded in the language, e.g., “It is hot here!” in a room with a window is likely to be interpreted as a request to open the window, while in a desert will be interpreted as a statement, and were *instructed to decide* whether they think the person wanted something from them or not (van Ackeren et al., 2012). Van Ackeren et al. found that the comprehension of IR sentences, as compared to sentences devoid of any implicit motor information, activated cortical motor areas as the left SMA and IPL bilateral, but not the M1 cortex. In another study by Moody and Gennari (2010), participants *read* the stimulus sentences describing actions requiring more or less physical effort, e.g., pushing the piano implies more physical effort than pushing the chair, and *occasionally answered* *comprehension* questions requiring a yes/no answer (e.g., did the man forget the piano?) by using their left hand when responding. The M1 cortex was not found among the regions activated by the items, while the premotor region was sensitive to the degree of effort implied by the actions.

On the other hand, there are several studies in which the M1 cortex has been reported among the regions activated by action words/verbs/sentences related stimuli. In on one of them, for instance, subjects (i) produced a verb corresponding to the presented noun (e.g., “drive” for “car”), and (ii) reading verbs and nouns (Frings et al., 2006). These authors found that among other areas, the M1 cortex was significantly activated during verb and noun silent *reading* task. In another study, *lexical decisions* about action verbs, i.e., to judge whether a verb is a real word or a pseudoword, led to stronger high-frequency EEG activity at recording sites located closely above primary motor (M1) cortex (Pulvermuller et al., 1999). If the processed action words are related to movements of different body parts, then the strongest in-going EEG current is detected close to the cortical representation of the respective body part (Pulvermuller et al., 1999). Interestingly, such a somatotopic activation of M1 has also been reported when participants *silently read* action words related to face, arm, or leg movements (Hauk et al., 2004) and even when they were presented with action words while they were engaged in a *distractor task* (Pulvermuller et al., 2005b). *Lexical decisions* activated the left sensorimotor area only for simple verbs with motor meanings and not for morphologically complex verbs built on a motor stem (e.g., comprehend, which contains the motor verb stem prehend; Ruschemeyer et al., 2007). Sub-threshold TMS stimulation of the hand area of left M1 leads to a facilitatory effect (i.e., faster response times in a *lexical decision* task) for arm- compared to leg-action-related words, and the opposite effect has been found for leg-action-related words after stimulation of the leg area (Pulvermuller et al., 2005a). The excitability of the left M1 hand area (as determined by supra-threshold stimulation and measured by motor evoked potentials, MEPs) is modulated during a *transformation task* involving action words as compared to non-action words (i.e., producing the singular/plural form of nouns or the third person singular/plural form for verbs; Oliveri et al., 2004). Similarly, *listening* to hand-action-related sentences decreased the amplitude of MEPs recorded from hand muscles, while listening to sentences related to foot actions modulated the MEPs recorded from foot muscles (Buccino et al., 2005). TMS delivered at the end of the sentence over the leg motor area in the left hemisphere caused larger MEPs recorded from the right gastrocnemius and tibialis anterior muscles during *silent reading* of legs related verbs included in literal, e.g., the man runs in the beautiful country, metaphorical, e.g., the woman runs with her fantasy often, and fictive motion sentences, e.g., the road runs along the impetuous river, than with idiomatic motion, e.g., between the neighbors runs bad blood, or mental sentences (Cacciari et al., 2011). Furthermore, *silent reading* of nouns referring to tools elicited activations in the hand area and silent reading of nouns referring foods elicited activation in regions implicated in mouth and face movements (Carota et al., 2012). Also, *passive* *silent reading* of hand verbs that described hand actions without tool-use, tool verbs, and their semantic radicals indicated hand involvement and tool verbs, and their semantic radicals indicated the tools or materials showed common activations within the hand-motion effect mask, in bilateral precentral gyrus (BA 4). *Silent reading* of idiomatic vs. literal sentences involving hand- and leg-related action words activated M1 when both idiomatic and literal sentences were being processed (Boulenger et al., 2009). A *go/no-go lexical tone judgment task* of Chinese tool-use action verbs emphasizing the hand involvement or the tool or material involvement and verbs that describe hand actions without tool-use in which participants were instructed to press button when the visually presented word had Tone 2 (low rising tone), activated within the motor localized mask precentral gyrus (BA 4) bilaterally for all three verb conditions (Yang and Shu, 2011). *Silent reading* of a series of sentences with a verb depicting either a mental state (e.g., deceive, persuade) or an action (e.g., punch, kick), and answering to a *comprehension* question that followed and required focusing on the mental state of a protagonist in half of the cases and the other half on actions involving a protagonist activated M1 (Kana et al., 2012), activated M1 (Kana et al., 2012). Interestingly, M1 was activated despite verbs being presented in a third singular person perspective, M1 was found activated in contrast with previous studies in which authors doubted whether they did not found M1 activation because they used the third person perspective (Gilead et al., 2013), consistently with a TMS study showing that motor simulation occurs for verbs in the first, but not in the third person perspective (Papeo et al., 2009). *Semantic generation* task, in which participants were instructed to quickly *describe how they would physically interact* with the visually presented pictures or words referring to objects that are typically used by hand or the foot, activated somatotopically M1 (Esopenko et al., 2012).

From the above mentioned literature it seems that it is neither the type of stimulus triggering M1 activation, since it appears clear that action-related words do not automatically activate the M1 cortex, nor the type of task, since it has been shown how, for instance, silent reading of or passive listening to action-related items might or might not activate the M1 cortex. This inconsistency of M1 activation may be explained with subjects performing or not performing mental simulation. These findings support our hypothesis that M1 activation depends on whether or not subjects choose to perform the motor imagery (explicitly or automatically) to solve the task requirements. If subjects use the strategy of simulating the movement referred to by the (action) verbs, M1 is activated; if, however, they use another strategy when solving the task at hand, M1 cortex is not activated. Consistent with this view, it has been shown that M1 cortex showed effector-specific activation for action hand verbs, as compared to non-manual actions (e.g., to kneel) during an imagery task in which participants were instructed to read the word, close their eyes, imagine performing the action, and open their eyes to indicate that they had finished motor imagery), but not during lexical decision (Willems et al., 2010). Willems et al. (2010) found that parts of PM distinguished manual from non-manual actions during both lexical decision and imagery, but there was no overlap or correlation between regions activated during the two tasks. Results from another study showed that unless explicitly instructed to perform mental imagery, M1 is not activated during language processing (Tomasino et al., 2007). A top-down modulation of strategies could determine whether participants do or do not perform mental simulation during language task. The motor imagery based strategy might be at work especially for tasks involving passive listening or passive silent reading and lexical decisions. According to this idea, in the above mentioned tasks involving action words (Pulvermuller et al., 2001, 2005a,b; Oliveri et al., 2004; Buccino et al., 2005; Tettamanti et al., 2005) subjects were free to use (or to refrain from using) the strategy of simulating the actions. The subjects’ free choice in underspecified task settings may explain why M1 is not always activated in the fMRI studies involving action word stimuli. As a consequence, the above mentioned results suggest that listening to or silent reading of action-related words items is not such a passive task as it is held. This view is supported by studies showing how the crucial factor that determines the activity in motor and premotor regions during action word processing seems to be that the context in which the word is presented. According to this view it has been suggested that the lack of M1 activation might be due to subjects not explicitly attending to the motor attributes of the words, raising the possibility that motor cortex modulation may occur only when participants directly attend to the actions and their motor properties (Kable et al., 2002, 2005). Cognitive studies suggest that language comprehension may not be based on a full word-by-word processing, and that the contextual meaning of the sentence may influence the semantic processing of the upcoming words (Marslen-Wilson and Tyler, 1980; Tyler and Wessels, 1983; Ferreira et al., 2002; Sanford and Sturt, 2002). Instructions too might be responsible for triggering or not a given processing strategy. It is known that cognitive processing of the same verbal stimuli can be modulated by explicit instructions (Fink et al., 2002). In the visuospatial domain, participants have been found to solve the Landmark test, both by explicitly comparing the lengths of the left and right line segments, and by computing the center of mass of the display. Solving the same task, by using the two strategies elicited different neural activations, with the explicit length comparisons (relative to line center judgments) differentially activating the left superior posterior parietal cortex, with a tendency toward activation of the equivalent area on the right, while the reverse comparison revealed differential activation in the lingual gyrus bilaterally and ACC (Fink et al., 2002).

Neuropsychological evidence supports the view of a top-down-dependent involvement of the sensorimotor cortex in linguistic processing. Neurosurgical patients with selective lesions of the precentral and postcentral sulci silently read action-related verbs (face-, hand-, and feet-related verbs plus neutral verbs) for subsequent (i) motor imagery by vividness ratings and (ii) frequency ratings. They showed a task × stimulus interaction: a lesion affecting a part of the cortex that represents a body part also led to slower RTs during the generation of mental images for verbs describing actions involving that same body part. By contrast, no category-related differences were seen in the frequency estimations (Tomasino et al., 2012). Two arguments have been put forward to rule out the possibility that sensorimotor activation during action words processing was due to secondary imaginary processes. In an attempt to minimize the influence of imagery, some authors administered the linguistic task first, followed by the action execution or observation tasks (Boulenger et al., 2006, 2009). Others suggested that the early neurophysiological activation spreading to M1 cortex revealed by MEG (Pulvermuller et al., 2005b) strongly speaks against the possibility that a second step imagery process is required. The motor activation occurs at about 150 ms after presentation of a written word, when normally lexical and semantic effects emerge (Pulvermuller et al., 2001, 2005a,b; Boulenger et al., 2006).

To establish when motor imagery exerts its influence over the sensorimotor activation, TMS has been applied at different points in time (Tomasino et al., 2008). Similarly to what has been found before (Pulvermuller et al., 2001, 2005a,b; Boulenger et al., 2006), a specific modulation of response times found as early as 150 ms. As a new feature, however, it has been clarified that the effect of the TMS selectively modulated the response times during the imagery task only, compared with the frequency judgment task and the silent reading task used as control conditions, suggesting that the effect of motor simulation occurs earlier (i.e., at 150 ms) than once thought (Pulvermuller et al., 2001, 2005a,b; Boulenger et al., 2006). This result is consistent with previous studies on motor imagery, showing that the activation of motor-related brain areas associated with motor imagery occurs very fast, within the first hundreds of milliseconds (Wang et al., 2010), and with evidence of sensorimotor activation as early as 270–390 ms after stimulus onset (Kawamichi et al., 1998). Lastly, similar results can be found in memorization of action sentences with an involvement of M1 detected between 150 and 250 ms after stimulus onset (Masumoto et al., 2006). In conclusion, we argue that an activation of M1 in word processing is comparable to what has been shown in the mental rotation literature with individuals solving the MR tasks by relying on different strategies. The view that people can use different strategies while processing action-related words hypothesizes that, in some circumstances, people understand action verbs/sentences in part by emphasizing motor representations of what it’s like to execute the designated action, in part by emphasizing visual representations of what it’s like to see the designated action. This view reinforces the parallel we are drawing between mental rotation and action word processing. As Taylor and Zwaan (2009) wrote to account for neuropsychological data on action-related word processing: “(…) comprehension relies on a multivariegated system for conceptual representation that relies on experiential memory (including motor, sensory, and intuitive experiential traces).” In addition, the top-down effect produced by the strategy use is strengthened now by neuroimaging evidence linking the visual-semantic motion features of action verbs/sentences with the left posterolateral temporal cortex (for a review, see Gennari, 2012). In this domain too it is held that modality-specific brain regions processing visual motion such as the middle temporal area or area V5 are not automatically or habitually engaged in language processing (Gennari, 2012). The lack of V5 activation in tasks in which motion information must be recruited suggests that V5 activation in is not integral to motion content processing *per se*, but rather it results from top-down influences or selective attention (Gennari, 2012). As it happens for the M1 cortex, the middle temporal area or area V5 is susceptible to top-down control and higher-level perceptual/conceptual influences: implied motion, apparent and illusory motion, “moving” sounds, and imagined motion can all elicit significant levels of activation in this area (Gennari, 2012). Similarly to M1 cortex, V5 responds more strongly when participants attend to motion compared to when they do not, even when the visual stimulation is the same (O’Craven et al., 1997).

Although it has been proposed that conceptual processing transcends the distinction between bottom-up, stimulus-driven, automatic processing, on the one hand, and top-down, strategy-driven, controlled processing, on the other hand (Simmons and Barsalou, 2003; Wilson-Mendenhall et al., 2012), the effect of strategy used during action-related verb processing might be still a promising approach.

## The Case of Non-Action Related, Negations, and Pseudo-Verbs Word Processing

The series of studies we have reviewed thus far clearly indicate that the activations in the sensorimotor areas, observed while participants are engaged in tasks involving non-action related words, and those observed while participants perform mental rotation of abstract stimuli (Kosslyn et al., 2001; Wraga et al., 2003) have a lot in common. Motor activity has been observed not only during action-related words processing, but also during reading imageable concrete words with no motor content (D’Esposito et al., 1997; Mellet et al., 1998; Pulvermuller and Hauk, 2006; Postle et al., 2008), “non-words” with regular phonology (Postle et al., 2008), and pseudo-verbs (Shapiro et al., 2005, see p. 1060; Tomasino et al., 2010b). It has been shown that non-motor related words and pseudo-verbs could activate (frontal) cortical areas to a similar extent as action-related verbs (see also Roder et al., 2002). Taken together these findings, in the measure in which they show that activation in sensorimotor areas is not selectively triggered by action-related word stimuli only, further weaken the bottom-up hypothesis which, on the contrary, speaks for a type of stimulus-dependent modulation of sensorimotor activation.

For instance, pseudo-verbs can activate motor areas, as it was shown in a fMRI study using a lexical decision task on positive and negative imperatives (Tomasino et al., 2010b). Importantly, these motor activations were not modulated by the linguistic context, in contrast to action-related verbs for which the motor activations were systematically modulated by positive and negative contexts. This result suggests that it is not the activation of the motor areas *per se* that allows distinguishing the effect of action verbs from that of pseudo-verbs, but rather the *systematic modulation* of the motor system activity by the linguistic context, which only occurs for action verbs. Importantly, similar unspecific activations of motor areas responses to “non-words” with regular phonology have been observed also in other studies (Hagoort et al., 1999; Postle et al., 2008).

Negations too have been found to both increase and decrease sensorimotor areas. Sentential negation has been argued to transiently reduce the access to mental representations of the negated information (Tettamanti et al., 2008). Indeed, it has been found that the activation in left fronto-parietal regions and the effective connectivity in concept-specific embodied systems are reduced in the case of action-related negative sentences (Tettamanti et al., 2008). Similarly, activations in the hand region of the primary motor and premotor cortices were found to be reduced for negative hand-action-related imperatives, such as “Don’t grasp!” compared to “Grasp!” (Tomasino et al., 2010b). Interestingly, the PM was also found to be activated, rather than reduced, by negations in other two studies involving a sentence-picture verification task (Hasegawa et al., 2002). According to the two-step simulation hypothesis of negation processing (Kaup and Zwaan, 2003; Kaup et al., 2007, 2010), when the comprehender processes negations, she creates a simulation of the negated state of affairs, and a simulation of the actual state of affairs. Negation is implicitly encoded in the deviation between both simulations (Ludtke et al., 2008). Taken together these results indicate that negations activate the sensorimotor cortex depending on whether the strategy of simulating the corresponding content of the sentences has or not been blocked. In Tomasino et al. (2010b), simulation was blocked by means of an experimental manipulation involving the use of imperatives known, if heard, to refrain the participants from performing the corresponding action. In a sentence–picture verification paradigm, they might be free to apply the two-step simulation strategy, leading to an activation of the sensorimotor areas. Negation processing thus constitutes a further piece of evidence of the top-down modulation of sensorimotor activations.

That motor representations are only engaged under specific conditions and their effects are context-dependent is also supported by studies in which idiomatic sentences or metaphors are used as stimuli. The activation of sensorimotor areas by metaphorical or idiomatic phrases – which convey abstract concepts embedded in concrete content – would support the theories that abstract concepts are understood through analogies to sensation and action (Lakoff and Johnson, 1980; Gibbs, 2006; Bergen, 2007). While Boulenger et al. (2009) found somatotopic activation for figurative and literal action sentences involving leg and arm verbs, other studies have yielded somewhat inconsistent results. For instance, Aziz-Zadeh et al. (2006) found a somatotopically organized activation in the PM cortex for literal action sentences, but not for idiomatic phrases, Raposo et al. (2009) too found an activation in the premotor/motor regions for isolated action verbs, and to a lesser extent for literal action sentences, but not for figurative sentences using action verbs. These findings lend support to cognitive theories of semantic flexibility, by showing that the nature of the semantic context determines the degree to which alternative senses and particularly relevant features are processed when a word is heard (Raposo et al., 2009).

The non-action related/abstract words are the last class of stimuli we will review here that, included in fMRI studies as a control condition, have been found to activate the sensorimotor areas. Embodied theories vary for the level of embodiment they assign to abstract concepts. The strong version of the embodied hypothesis holds that abstract concepts, just like concrete ones, are grounded in the sensorimotor system (Lakoff and Johnson, 1980; Glenberg et al., 2008). Others have proposed that abstract and action-related word processing reflects a continuum rather than a dichotomy (Scorolli et al., 2011) since in a rating study about concreteness judgments on large sets of words a bimodal distribution (according to features, such as tangibility or visibility of the items), was found (Nelson and Schreiber, 1992). Evidence in support of the stronger version of embodiment is shaky. In fact, abstract sentences (e.g., to give some news) may (Glenberg et al., 2008) or may not (Ruschemeyer et al., 2007) exactly activate motor information as concrete ones do (e.g., to give a pizza). By comparing simple action-related verbs [such as “greifen” (to grasp)] and complex abstract verbs [such as “begreifen” (to comprehend)], Ruschemeyer et al. (2007) showed that only the former, triggered activity in premotor areas. Similarly, Tettamanti et al. (2005) reported a selective activation of motor areas for concrete sentences containing a manipulable object as opposed to sentences containing abstract objects.

Here we propose that the activation of the sensorimotor areas in association with abstract stimuli is most likely due to the intervention of mental imagery. Implicit motor imagery is not uniquely used when a body-related verb stimulus is encountered, and might be defined as a strategy implicitly triggered in association to generic imageable words, and proved adequate for eliciting activity in motor areas (Postle et al., 2008). The selected strategy can be implicitly transferred from one stimulus to another. In Wraga et al.’s (2003) study, while one group of participants saw a MR of hands block followed by a MR of 3D cubes block, a different group saw two sets of MR of 3D cubes blocks. They found that the left M1 cortex, the left insula, and the PM area bilaterally were selectively activated in participants who performed the MR of hand shapes before the MR of 3D cubes. By contrast, the right superior parietal lobe and the right occipito-temporal junction were enhanced in participants who performed only the MR of 3D cubes. The authors concluded that the motor strategy can covertly be transferred to the imagined transformations of stimuli other than body parts such as abstract ones. In a recent fMRI study, in which a similar implicit transfer of strategies paradigm was applied to motor and non-motor related verbs processing (Papeo et al., 2012), it was examined whether motor strategies adopted during a motor imagery task creates a cognitive context that would be implicitly transferred to a subsequent linguistic task. Participants performed a mental rotation block of either motor or visuospatial strategy, randomly presented before each block of silent reading of verbs describing hand actions or physical/psychological states. Irrespective of the verb category, reading following a mental rotation block of motor strategy, compared to reading following a mental rotation block of visuospatial strategy, increased activity in left primary motor cortex, bilateral PM and right somatosensory cortex. Thus, the cognitive context induced by the preceding motor strategy-based mental rotation modulated word-related sensorimotor responses. In a recent TMS study of the left M1 cortex (Scorolli et al., 2012; non-idiomatic), phrases composed by abstract or concrete verbs combined with abstract or concrete nouns (AA, CA, AC, CC) have been used. The authors found an early motor activation with concrete verbs and a delayed one with abstract verbs. This result first confirms the view that abstract words (verbs) also activate the motor system related to manual action. In addition, as to the delayed activation, authors argue that it is likely that the effort to process abstract words in the premotor cortex or other secondary areas is higher and therefore determines a stronger modulatory influence on M1.

With respect to the possible transfer of strategy account, as in this paradigm the context is induced by both action-related or non-action related verbs, with combinations of abstract verbs plus (abstract or concrete) nouns, the putative effect of transfer would be attenuated. Nevertheless, one cannot exclude that a preceding block, in which concrete verbs and concrete nouns were combined (e.g., grasp a pen), might have favored the transfer of a (motor) strategy effect on the subsequent block of concrete verb plus abstract noun, e.g., grasp an idea; or that a preceding block in which abstract verbs and concrete nouns are combined (e.g., suspect a pen), might have prompted a transfer of (motor) strategy effect, in this case triggered by the noun, on the subsequent block of abstract verb plus abstract noun, e.g., suspect freedom (i.e., non-sensible phrases). The results indeed showed greater MEPs amplitude for non-sensible phrases containing concrete verbs followed by abstract nouns).

Furthermore, as the timing of TMS is known to modulate action word processing (Papeo et al., 2009), one cannot exclude that an interaction between a putative transfer of strategies effect and stimulation time occurred in Scorolli et al.’s study. Showing that words with an abstract content can too enhance the sensorimotor areas activation strongly implies that the type of stimulus does not automatically trigger motor simulation as the embodied hypothesis would predict.

## Conclusion

To wrap up, in the case of both mental rotation and action word processing, motor simulation is not automatically triggered by the type of stimulus but by the type of strategy. We then argued that the type of strategy selected depends on top-down modulation such as the context and tasks demands. We also argued that whether the sensorimotor cortex is or it is not activated is determined by the type of strategy selected in word processing. Thus, the motor simulation is neither automatic nor necessary to language understanding. The top-down hypothesis instead holds that motor activation is not automatically triggered by the type of stimulus but by the type of strategy. Also the embodied cognition hypothesis, as it claims that “understanding” *is* sensory and motor simulation, is not compatible with the view that the type of strategy selected depends on top-down modulation of the context and tasks demands. Rather the top-down hypothesis is in line with the disembodied view the motor system may be activated but not necessarily so (Mahon and Caramazza, 2005, 2008).

Our view is consistent with the notion of flexibility in language representation whereby the degree to which a modality-specific region contributes to a representation depends on the context (Hoenig et al., 2008; van Dam et al., 2010b, 2012) in which conceptual features are retrieved. Flexibility is characterized by the relative presence or absence of activation in motor and perceptual brain areas. The key idea is that words are associated with more than one experiential feature; accordingly, word processing could be modified by encouraging participants to focus on one propriety vs. another. We also add that this top-down modulation might exert its influence also in selecting the type of strategy adopted while processing language. Our preferred view is that, as it happens in the mental rotation domain, neither the type of stimulus nor the type of task seems to automatically trigger M1 activation. Rather we propose that different strategies will cause participants to lean on different sorts of sensorimotor representations. According to this view M1 activation depends on whether or not subjects choose motor imagery (explicitly or automatically) as a strategy to solve the task requirements. The subjects’ free choice in task settings may explain why M1 is not always activated in the fMRI studies involving action word stimuli. Particularly relevant here is the result that neural activity in M1 cortex areas 4a and 4p seems to be differentially modulated by attention to action (Binkofski et al., 2002). Accordingly, it has been suggested that the lack of M1 activation might be due to subjects not explicitly attending to the motor attributes of the words, thus raising the possibility that motor cortex modulation may occur only when participants directly attend to the actions and their motor properties. Lastly, this view is in accordance with studies suggesting that a crucial factor for observing activity in motor and premotor regions during action word processing seems to be that the context in which the word is presented supports a motor interpretation and that the word form as a whole conveys a motor meaning (van Dam et al., 2012).

## Conflict of Interest Statement

The authors declare that the research was conducted in the absence of any commercial or financial relationships that could be construed as a potential conflict of interest.
